# Computer-assisted versus non-computer-assisted preoperative planning of corrective osteotomy for extra-articular distal radius malunions: a randomized controlled trial

**DOI:** 10.1186/1471-2474-11-282

**Published:** 2010-12-14

**Authors:** Natalie L Leong, Geert A Buijze, Eric C Fu, Filip Stockmans, Jesse B Jupiter

**Affiliations:** 1 Massachusetts General Hospital, Department of Orthopaedic Surgery, 55 Fruit Street, YAW-2-2C, Boston, Massachusetts 02114, USA; 2 Handgroep Groeninge, AZ Groeninge, Kortrijk, Belgium

## Abstract

**Background:**

Malunion is the most common complication of distal radius fracture. It has previously been demonstrated that there is a correlation between the quality of anatomical correction and overall wrist function. However, surgical correction can be difficult because of the often complex anatomy associated with this condition. Computer assisted surgical planning, combined with patient-specific surgical guides, has the potential to improve pre-operative understanding of patient anatomy as well as intra-operative accuracy. For patients with malunion of the distal radius fracture, this technology could significantly improve clinical outcomes that largely depend on the quality of restoration of normal anatomy. Therefore, the objective of this study is to compare patient outcomes after corrective osteotomy for distal radius malunion with and without preoperative computer-assisted planning and peri-operative patient-specific surgical guides.

**Methods/Design:**

This study is a multi-center randomized controlled trial of conventional planning versus computer-assisted planning for surgical correction of distal radius malunion. Adult patients with extra-articular malunion of the distal radius will be invited to enroll in our study. After providing informed consent, subjects will be randomized to two groups: one group will receive corrective surgery with conventional preoperative planning, while the other will receive corrective surgery with computer-assisted pre-operative planning and peri-operative patient specific surgical guides. In the computer-assisted planning group, a CT scan of the affected forearm as well as the normal, contralateral forearm will be obtained. The images will be used to construct a 3D anatomical model of the defect and patient-specific surgical guides will be manufactured. Outcome will be measured by DASH and PRWE scores, grip strength, radiographic measurements, and patient satisfaction at 3, 6, and 12 months postoperatively.

**Discussion:**

Computer-assisted surgical planning, combined with patient-specific surgical guides, is a powerful new technology that has the potential to improve the accuracy and consistency of orthopaedic surgery. To date, the role of this technology in upper extremity surgery has not been adequately investigated, and it is unclear whether its use provides any significant clinical benefit over traditional preoperative imaging protocols. Our study will represent the first randomized controlled trial investigating the use of computer assisted surgery in corrective osteotomy for distal radius malunions.

**Trial registration:**

NCT01193010

## Background

Despite advances in internal fixation devices, malunion of distal radius fractures is still the most common complication after wrist fractures, with overall malunion rates as high as 17% [1]. Patients with malunion of the distal radius can experience significant disability in the form of pain, arthritis, decreased range-of-motion, weakness, and visible deformity [2-7]. For those who undergo operative treatment, clinical studies have demonstrated a positive correlation between a more accurate anatomic correction and eventual overall wrist function [8-11]. However, surgical correction presents a challenge to orthopedic surgeons because of the complex anatomic deformity often associated with this condition; accurate preoperative planning is crucial for surgical success. Standard radiographs are often sufficient for simple deformities in the coronal or sagittal planes; however, the majority of distal radius fracture malunions have more than one plane of deformity [12-14]. Reformatted 3D CT reconstructed models have improved the surgeon's ability to conceptualize the multiple planes of deformity in more complex malunions; however, these models still only serve as visual templates for intra-operative referencing.

New virtual surgical planning technology that combines CT imaging and state-of-the-art software has recently been developed. To date only small case series and a case report using this technology for corrective osteotomy of malunited distal radius fractures have been published [13,15-18]. Although these studies report promising results, existing methodologies have not fully utilized available technological capabilities and there has been no study to date that compares patient outcomes using these computer assisted preoperative planning techniques to conventional pre-operative planning techniques for surgical correction of malunited distal radius fractures.

In order to understand the role that virtual surgery techniques should play in corrective osteotomy of malunited distal radius fractures, it is critical to understand how using this technology compares to conventional pre-operative planning in terms of functional outcomes, patient satisfaction, and pain.

The objectives of this study are to compare patient outcomes after corrective osteotomy for malunited distal radius fractures with and without preoperative computer-assisted planning and patient-specific surgical guides.

## Methods/Design

### Participants

All patients older than 18 years of age undergoing elective surgery for symptomatic malunited extra-articular distal radius fractures by the surgeons participating in this study will be invited to participate. Subjects will be invited to enroll during their routine preoperative office visit for care of their wrist. The protocol will be explained in detail and informed consent obtained prior to the initiation of any treatment. Patients will be given a copy of the consent form, and be informed that their participation is voluntary and that they can withdraw at any time. After discussing the risks/benefits and alternatives to participation, the patient will sign the consent form.

#### Inclusion criteria

• Adult patient (age 18 years or greater)

• Extra-articular malunion of the distal radius, following the criteria for malunion as defined by McQueen et al. [19] as one of the following compared with the opposite normal side:

○ dorsal tilt >10°

○ volar tilt >15°

○ radial shortening >3 mm

• Indications for osteotomy are pain, weakness, decreased palmar flexion, incongruency of the DRUJ and adaptive carpal instability

• At least 3 months post-injury

• Fluent in English

#### Exclusion criteria

• Intra-articular malunion with a step-off or gap >1 mm

• Associated injuries of the ipsilateral forearm

• Functional disability for any other reason than the malunion

• Pathology of the contralateral forearm

• Patients with impaired decision-making capacity

• Pregnancy

• Prisoners

### Intervention

**Figure 1:**
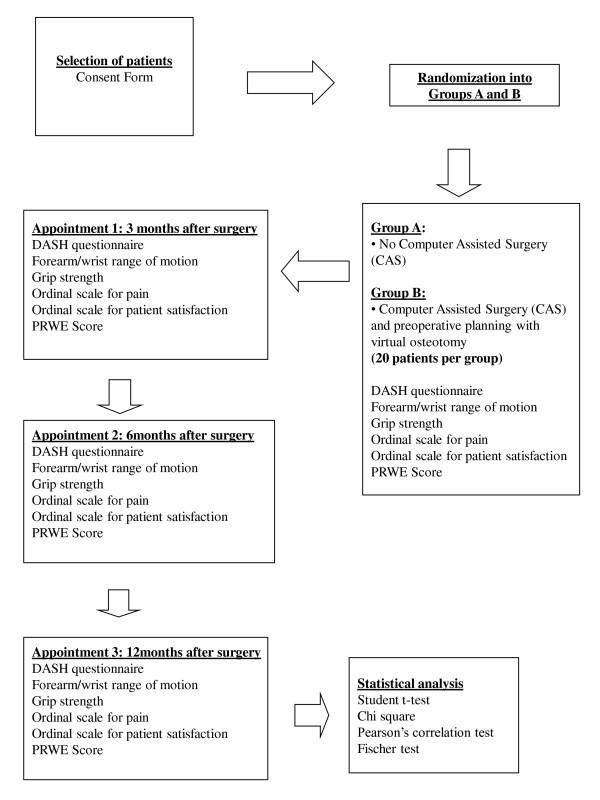
**Flux scheme for study**.

This study is designed as a randomized controlled trial, comparing two groups of patients with symptomatic extra-articular malunited distal radius fractures (Figure 1). One group of patients will undergo corrective surgery of the distal radius, with preoperative computer-assisted planning and virtual osteotomy, and the other group will undergo corrective surgery, with conventional (non-computer-assisted) preoperative planning. In both groups, the surgeons will be restricted to using a volar plate as the fixation device for the osteotomy. Also, in both groups, if inadequate cortical apposition is obtained intra-operatively, bone graft will be used.

#### Computer-assisted planning

##### CT imaging and 3D Forearm Model Reconstruction

The first step will be to obtain CT images of both the malunited and the contralateral normal forearm. The forearms will be scanned using high-resolution axial plane images with the forearms in neutral position. The CT images will be acquired using standard clinical scanners available at each participating institution. The data obtained will consist of parallel digital images, each with a thickness of 0.625 mm, and a resolution of 512 × 512 pixels. The CT images of the forearm and wrist will be exported to a surgical planning company (SurgiCase Orthopaedics, Materialise, Leuven, Belgium) to construct a 3D anatomical model.

##### Virtual osteotomy

Surface-rendered bony models from the CT images will be created, and the virtual osteotomy will be performed. In our study, software will be used to reposition and align the osteotomy fragments to best fit the uninjured side (which will serve as a template for normal anatomy). After the osteotomy fragments have been fitted to the uninjured template, the quality inspection software will identify areas of inconsistent overlap and quantify the distance of separation between the two models. After repositioning, a virtual 3D model of the distal radius will be created.

##### Surgical guides

Two synthetic patient specific surgical guides will be manufactured (SurgiCase Orthopaedics, Materialise, Leuven, Belgium) and sterilized for use as drill and saw guides in the operating room. These drill guides are a 3D synthetic model of the radius with the position of the cut indicated will be provided to the surgeon for the procedure. The guides will be designed for use with standard locking compression plates.

##### Osteotomy Cut, Synthetic Template Spacer, and Fixation

**Figure 2:**
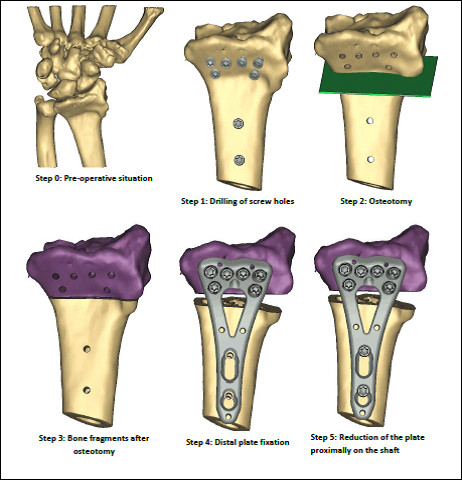
**Volar view of 3D reconstruction of distal radius malunion (Step 0) and the surgical procedure (Steps 1-5) that will be performed with computer-assisted planning**. Two temporary drill guides (not shown) will be used to determine the placement of the drill holes and the location of the osteotomy cut.

First, the fit of the two surgical guides will be tested against the distal radius for fit. To hold the first guide in place, 1.25 m K-wires will be drilled into the small fixation holes that correspond with the temporary fixation holes on the guide. Screw holes will be drilled in the distal radius using surgical guide 1, along with metal drill guide 312.181 from Synthes. Then, surgical guide 1 will be removed, and surgical guide 2 will be fixed to the bone using K-wires, in the same fixation holes previously drilled. The osteotomy will be performed with the assistance of surgical guide 2. Next, the locking compression plate (LCP) will be fixed onto the distal bone fragment. Then, the plate will be fixed proximally onto the radial shaft. A 2.4 mm variable angle LCP two-column volar distal radius plate from Synthes, 2.4 mm/1.8 mm drill guide from Synthes, 1.25 mm K-wires, and 0.4 mm thick saw will be required for the procedure, in addition to the surgical guides provided by Materialise (Figure 2).

#### Conventional planning

Planning for the operations will be performed as usual by the surgeon, using the mirrored contralateral contour as the reconstructive goal. The planning is performed with a volar approach and the intention is to use a volar plate as a fixation device after the osteotomy. Imaging for pre-operative planning will be restricted to the standard plain radiographic views that are currently the standard of care.

After surgery, for both groups, the types of exercises and hand therapy used and the timing of their introduction will be standardized according to existing protocols that the Occupational Therapy Service has developed in conjunction with the Orthopaedic and Plastic Surgical Hand Services. One week after surgery, all study subjects will be contacted by phone by research staff to rule out any adverse post-operative events such as infection or undue pain. Outcome will be assessed at 3, 6, and 12 months after surgery during regular follow-up visits.

##### Objectives

The objective of this study is to compare patient outcomes after corrective osteotomy for malunited distal radius fractures with and without preoperative computer-assisted planning and patient-specific surgical guides. Our null hypotheses are that there is no difference between computer-assisted surgical planning and conventional surgical planning in terms of 1) functional outcome, 2) radiological outcome, and 3) economical analysis.

##### Outcomes

Functional outcome will be measured by both patient and physician-rated outcomes. Patient-rated outcomes will include the Disability of the Arm, Shoulder, and Hand (DASH) score [20], Patient Rated Wrist Evaluation (PRWE) score [21], as well as patient satisfaction and pain 10-point ordinal scales. Physician-rated outcome will be measured by bilateral range-of-motion (volar flexion, dorsal flexion, supination, pronation, radial deviation, ulnar deviation, all measured by a goniometer), bilateral grip strength (measured by grip dynamometer). Range of motion and grip strength will be performed with the elbow flexed at 90°, using a correctly calibrated JAMAR dynamometer, taking care to ensure that the subject is not leaning on the table.

Radiographic outcome will be measured on on standard PA and lateral X-rays. This will include volar angulation (°), radial inclination (°), ulnar variance (mm), and articular incongruity (mm) [22].

In both groups, the total time from surgical incision to closure will be recorded, and the total time of fluoroscopy use will be recorded.

All outcome measures will be collected preoperatively by the treating physician and/or a research assistant, and postoperatively by an independent individual.

##### Sample Size

Patient-rated functional outcome, as measured by DASH scores, is considered the most important outcome measure for patients with distal radius fractures. Our power analysis revealed that in order to detect a difference in DASH scores between the two groups with a 80% power, α = 0.05, and an estimated sigma = 10 degrees, 17 subjects will be required in each group. In order to account a lost to follow-up rate of approximately 15%, it is our goal to enroll 20 patients in each group.

##### Randomization

A random binary sequence of was generated by our research fellow (Microsoft Excel), with 0 corresponding to the control group and 1 corresponding to the experimental group. The sequence is concealed from participating surgeons. After a patient is enrolled in the study by the participating surgeon, the surgeon will email a central research coordinator, who will then assign the next number in the sequence to the study subject and inform the surgeon of the allocation. There will be no blinding. The study participants, surgeons, and other members of the research team will all be aware of the intervention group to which the participants are assigned.

##### Statistical Methods

All data will be tested for normality (Gaussian-shaped distribution) using the Komogorov-Smirnov test and homogeneity of variant by Bartlett's test. ANOVA and Tukey tests will be used for variables conforming to a normal distribution. Otherwise, nonparametric procedures (including the Kruskal-Wallis and Wilcoxon tests) will be used. For all statistical tests, differences where p < 0.05 (two-tailed) will be considered significant. The SPSS statistical package will be used for the analysis of the data (version 15.0, SPSS Inc., Chicago, IL). The power and sample size calculations have been performed with use of the nQuery Advisor software program (version 5.0, Statistical Solutions, Boston, MA).

This study will be reported according to CONSORT guidelines [23].

##### Ethics

This study conforms to the Declaration of Helsinki regarding ethical principles for research with human subjects, and is not in violation of any local laws. This study has been approved by the Partners Healthcare Institutional Review Board (IRB), the ethical governing body for Massachusetts General Hospital. It has also been approved after full review by the University of Louisville Institutional Review Board, the ethical governing body for the Kleinert Kutz Hand Care Center. IRB approval is pending at other study sites, which have not yet begun subject enrollment.

## Competing interests

NL, GB, EF, and JJ have no financial or non-financial competing interests to declare. FS serves as a clinical advisor for Materialise N.V.

## Authors' contributions

NL and GB drafted this manuscript, participated in study design, and participated in study coordination. EF performed background research for this study and contributed to study design. FS participated in study coordination and supervision of the computer assisted planning. JJ conceived of this study, participated in study coordination, and participated in study design. All authors read and approved this manuscript.

## Pre-publication history

The pre-publication history for this paper can be accessed here:

http://www.biomedcentral.com/1471-2474/11/282/prepub
